# “It’s just a fever”: Gender based barriers to care-seeking for visceral leishmaniasis in highly endemic districts of India: A qualitative study

**DOI:** 10.1371/journal.pntd.0007457

**Published:** 2019-06-27

**Authors:** Beulah Jayakumar, Nirmala Murthy, Kingsuk Misra, Sakib Burza

**Affiliations:** 1 Consultant, New Delhi, India; 2 Foundation for Research in Health Systems, Bangalore, India; 3 KalaCORE, New Delhi, India; Institut Pasteur, FRANCE

## Abstract

**Introduction:**

Diagnosis and treatment for visceral leishmaniasis (VL) is considered to be delayed amongst poor, rural women in highly endemic districts of Bihar and Jharkhand. The objective of this study was to assess and understand barriers to VL diagnosis and treatment for women in endemic districts with a high burden of VL.

**Methods:**

The study used a stratified and purposive sample of 33 female patients with VL, 11 health staff, 11 local (unqualified) health providers and 12 groups of community elders drawn from ten districts in Bihar and four in Jharkhand with high burdens of VL. The study was conducted within an exploratory and inductive framework, using semi-structured in-depth interviews and discussions.

**Results:**

Women accessing treatment more quickly tended to move faster from treating their symptoms on their own to seeking care from local providers. Perception among female patients of the illness being not serious (owing to initially non-specific and mild symptoms), lack of money, prioritisation of household chores over their need to seek care and the absence of a male guardian to accompany them in seeking care at facilities worked together to drive these choices. Most patients and their families did not suspect VL as the cause for their non-specific symptoms, but when VL was suspected, treatment shopping ended. Lack of prioritization of women’s health issues appears to be a pervasive underlying factor. Public health facilities were not an early treatment choice for the majority, but where it was, the diagnosis of VL was often not considered when presenting with under 2 weeks of symptoms, nor were appropriate follow-up plans instituted.

**Conclusion:**

The insidious presentation of VL and the low prioritisation of women’s health need to be jointly addressed through messages that emphasise the importance of early diagnosis and treatment of disease, which is low-cost in time and money when managed in public health facilities. Clear messages that project prioritising women’s care-seeking over household work as a smart choice and the need for rallying male support are needed. Additionally, efforts to reduce missed opportunities through early case suspicion and engaging private providers to better counsel women with suspected VL could close critical gaps in the continuum of care.

## Author summary

India bears the greatest burden of a fatal parasitic disease called visceral leishmaniasis (VL), popularly known as Kala Azar. The disease is confined mostly to hot spots in Bihar and Jharkhand in the eastern part of the country. Amongst factors hampering efforts to eliminate VL are delays from the onset of symptoms to the time patients are diagnosed and treated, where disease transmission is thought to be greatest and longer delays result in poorer outcomes; this is considered to be a particular problem in poorer women living in rural areas. This study found patterns in careseeking: initial self-medication followed by multiple visits to local (unqualified) providers and visits to private facilities, before ultimately reaching the public health facilities where treatment was freely available. Those who did attend public health facilities early in their illness were not tested for VL nor followed up with a possible diagnosis of VL in mind. Female patients tended to under-estimate the severity of their illness, while social and economic reasons also influenced care-seeking behaviour–particularly the lack of a male relative in the house, and a reluctance to utilise already meagre resources. The conclusions of the study were the need to encourage women with persistent fever to seek care without delay, while ensuring that factors that serve as barriers, such as low prioritisation of women’s health by households (and the women themselves) is countered by messages emphasizing the danger of delayed treatment for VL and the low time-cost burden of availing care within the public health sector. Improving awareness of VL amongst informal and formal health providers remains key in this process.

## Introduction

Visceral leishmaniasis (VL) or Kala azar, is a vector-borne disease caused by the protozoan *Leishmania donovani* and transmitted through the bite of the phlebotomine sand fly. Up to 100,000 cases are estimated to occur globally every year [1], and the disease is normally fatal within two years if untreated [2]. The pathogenesis of VL is complex and clinical presentations vary from asymptomatic infection to fatal disease [3,4].

While VL is highly endemic in the Indian subcontinent and East Africa, India accounts for nearly half of all cases worldwide [1]. The epidemiological features of the disease in the Indian subcontinent together with the availability of effective diagnostic, treatment and vector control measures make it amenable to its elimination as a public health problem [5]. As such, since 2005, India has been part of a regional initiative to achieve this elimination target [6], defined as less than 1 case per 10,000 population at the sub-district level. In the Indian context, these units are called blocks, with populations ranging from 80,000 to 300,000. As a result of the elimination efforts, and possibly also due to the cyclical epidemic nature of VL, there has been a steady decline in reported cases, with an 82% decline between 2011 and 2017 [7].

Despite the encouraging trend, much remains to be done. India remains the only country in the regional elimination initiative that has not yet reached the threshold target, with the disease continuing to be endemic in the states of Bihar, Jharkhand, West Bengal and Uttar Pradesh. By 2018, 49 blocks in Bihar and 25 blocks in Jharkhand have not yet achieved the elimination threshold, with Bihar contributing to nearly 70% of national cases [7]. These highly endemic blocks are spread across 10 districts in Bihar and four districts in Jharkhand.

Under the aegis of the National Roadmap for Kala azar Elimination and with support from a range of stakeholders, over recent years there has been an accelerated effort to control the disease, with rapid diagnostic tests made available at all, and treatment with single-dose Liposomal Amphotericin B at selected nodal public health care facilities in endemic blocks [8]. Kala azar technical supervisors (KTS) at each block oversee vector management and active surveillance, while frontline health workers called Accredited Social Health Activists (ASHA) support prevention and case detection activities at the community level. Development partners have also been conducting a wide range of community education efforts for VL.

Timely detection and treatment of those with acute symptomatic VL is the mainstay of elimination efforts, as this results in the interruption and shortening of transmission from human hosts, which are currently the only known reservoir in the Indian subcontinent. Results from recent modelling studies suggested that decreasing the time between the onset of symptoms to treatment (OT) from 40 to 20 days could bring the elimination target forward by a year [9], with an even larger potential in Bihar [10].

Women with VL appear to access care later than men. A retrospective study of VL patient data from 2012–13 in Bihar found significantly lower proportion of women among reported cases compared to the background population. The paper concluded that this is likely due to under-reporting (as a result of poorer access to healthcare for women), and not necessarily due to lower incidence amongst women, as there was no corresponding difference between the sexes among those under 15 years of age [11]. This is supported by a 2014 study that showed a marked decrease in the proportion of female patients with rising age [12], while an earlier 2006 study that showed that 60–80% of VL patients in facilities were men [13]. A 2003 study from Bangladesh reported that in one highly affected village, reproductive-age women were three times as likely to die from VL compared to men or children; where VL accounted for 23% of all deaths, 80% of these were adult women. Qualitative data from the same study suggest that women experienced substantial barriers to seeking care [14].

VL remains a disease of the poor, with 83% households belonged to the lowest two wealth quintiles in the state’s wealth distribution, while 70% live in mud adobe houses, consistent with the breeding preferences of the sand fly [15]. Caste is an important and reliable indicator of socio-economic status and is used in national surveys as a measure of economic inequality and access to services. It appears that those belonging to the caste categories of Scheduled Caste (SC), and Scheduled Tribes (ST) are disproportionately impacted by VL: a 2012 study of VL patients in Bihar, found that patients from SC had twice the odds of presenting late at treatment centres than others [16].

The objective of this study was to assess and understand barriers to VL diagnosis and care for women in endemic districts with a high burden of VL.

## Methods

### Study design

The study was designed to be exploratory and inductive within the post-positivist paradigm, using qualitative inquiry techniques. A purposive and stratified sample was used, with the overall intent of providing a rich and contextualized understanding of care-seeking patterns and drivers of those choices of women patients, but also to serve as an *ex-ante* strategy for extrapolating results towards providing evidence for practice. The days between onset of symptoms to treatment (OT) was used to stratify the sample into:

- Women with OT < 28 (early access, ‘WE’)
- Women with OT 28–50 (moderate access, ‘WM’)
- Women with OT >50 (late access, ‘WL’)

Other participants in the study were:

- Family member of those with confirmed VL who had died before treatment
- Kala azar technical supervisors (KTS)
- Local, unqualified healthcare providers (local providers)
- Community members of locations where women with moderate or late access lived

In addition, a smaller sample of male patients with VL with OT of 28–50 days and > 50 days was selected and investigated as a form of quality control, to compare the findings related to female patients and thus remain open and alert to alternative explanations for themes that emerge for female patients.

### Sampling strategy and sample size

The total sample size was a trade-off between that which would sufficiently answer the research question and capture a range of experiences, without being too repetitive, and what was feasible. Sampling for patients with VL was done in three stages. In the first stage, 12 blocks were selected as study sites using the Kala Azar Management Information System (KAMIS) national surveillance data from 2015–17, based on the relative distribution of the disease burden between Bihar and Jharkhand. Two blocks each from four of the 10 highly endemic districts in Bihar and one each from all four endemic districts in Jharkhand were selected, using the following criteria:

- Blocks above the elimination threshold of one case per 10,000 population in 2016
- Blocks with the highest incidence of VL per 10,000 population within their respective district
- Blocks with the longest median OT amongst those registered between January and August 2017, and
- Blocks with the largest number of cases with OT >50.

Of the 12 blocks selected, all met the above criteria aside from one, which met the first three but did not have cases with OT>50, which was selected for logistical reasons. S1 Table gives the details of selection criteria listed above for the blocks that were selected for the study.

In the second stage, a list of patients with VL aged 18 years and above were obtained from the selected blocks and organized by age, caste and OT, and samples were chosen for each of the OT categories of female patients, ensuring those belonging to SC (in Bihar) and those belonging to ST (in Jharkhand) were included. Additional samples were selected as backup. One male patient–with either moderate or late access–was selected from nine of the twelve blocks chosen.

In the third stage, field teams obtained identifying information of these pre-selected patients from the surveillance register maintained at the block level by the KTS, along with details of those with confirmed VL who had died before treatment. Where the pre-selected patients were not traced, new cases from September–December 2017 were included. The final sample included 45 patients: 10 women with early access, 10 women with moderate access, 13 women with late access, 3 men with moderate access, 6 men with late access and 3 who died from confirmed VL. The deceased include two men and one male child, and interviews were conducted out with the wives of the male patients and the mother of the child. In addition, the following non-patient participants were selected: 11 KTS, 11 local providers and groups of community members in 12 communities. Table 1 gives the distribution of the sample across study units:

**Table 1:** Sample size and distribution.

State	District	Block	Female Patients			Male “Control” Patients		FGD	Local providers	KTS	Dead from VL
			Women with early access (OT<28)	Women with moderate access (OT 28–50)	Women with late access (OT >50)	Men with moderate access (OT 28–50)	Men with late access (OT >50)				
Bihar	E Champaran	Madhuban	1		2		1	1	1	1	
Bihar	E Champaran	Chakiya	1	1	1		1	1	1	1	
Bihar	Sitamarhi	Dumra	1	1		1		1	1	1	
Bihar	Sitamarhi	Pupri	1	3				1	1	1	
Bihar	Gopalganj	Gopalganj Sadar	1		2	1		1	1	1	
Bihar	Gopalganj	Bharauli	1	1	1		1	1	1	1	
Bihar	Araria	Raniganj	1		2		1	1	1	1	
Bihar	Araria	Forbesganj	1	2		1		1	1	1	1
Jharkhand	Dumka	Gopikander	1		1		1	1	1	1	1
Jharkhand	Pakur	Maheshpur	1	1	1		1	1		1	1
Jharkhand	Godda	Boarijore			2			1	1		
Jharkhand	Sahibganj	Mandro		1	1			1	1	1	
**Totals**	** **	** **	**10**	**10**	**13**	**3**	**6**	**12**	**11**	**11**	**3**

### Data collection

Field teams carried out in-depth interviews using field-tested semi-structured guides that probed for factors affecting care-seeking, circumstances around each decision and sought to construct a timeline for care-seeking. Similar tools were used to carry out in-depth interviews of the KTS, local providers and focus group discussions in communities to explore trends in care-seeking patterns and their perspectives on factors affecting decisions about care-seeking. Data collection teams consisted of an interviewer/facilitator, note-taker and a manager. Data was collected between December 2017 and January 2018.

### Data processing and analysis

Transcribed data and field notes were translated to English and coded in NViVo 8, along with analytic reflective notes of the principal investigator, forming and refining categories as the coding progressed. Data was further compared and contrasted to discern conceptual similarities as well as outliers and refine the distinctions between categories until a theoretical framework emerged.

### Ethics statement

The study was approved by the Ethics Review Committee of the Foundation for Research in Health Systems, Bangalore, India. Written informed consent was obtained from all participants in the study. Standard procedures for maintaining patient confidentiality and data privacy were followed. All human subjects were 18 years of age or older.

## Results

### Background characteristics

**Fig 1:**
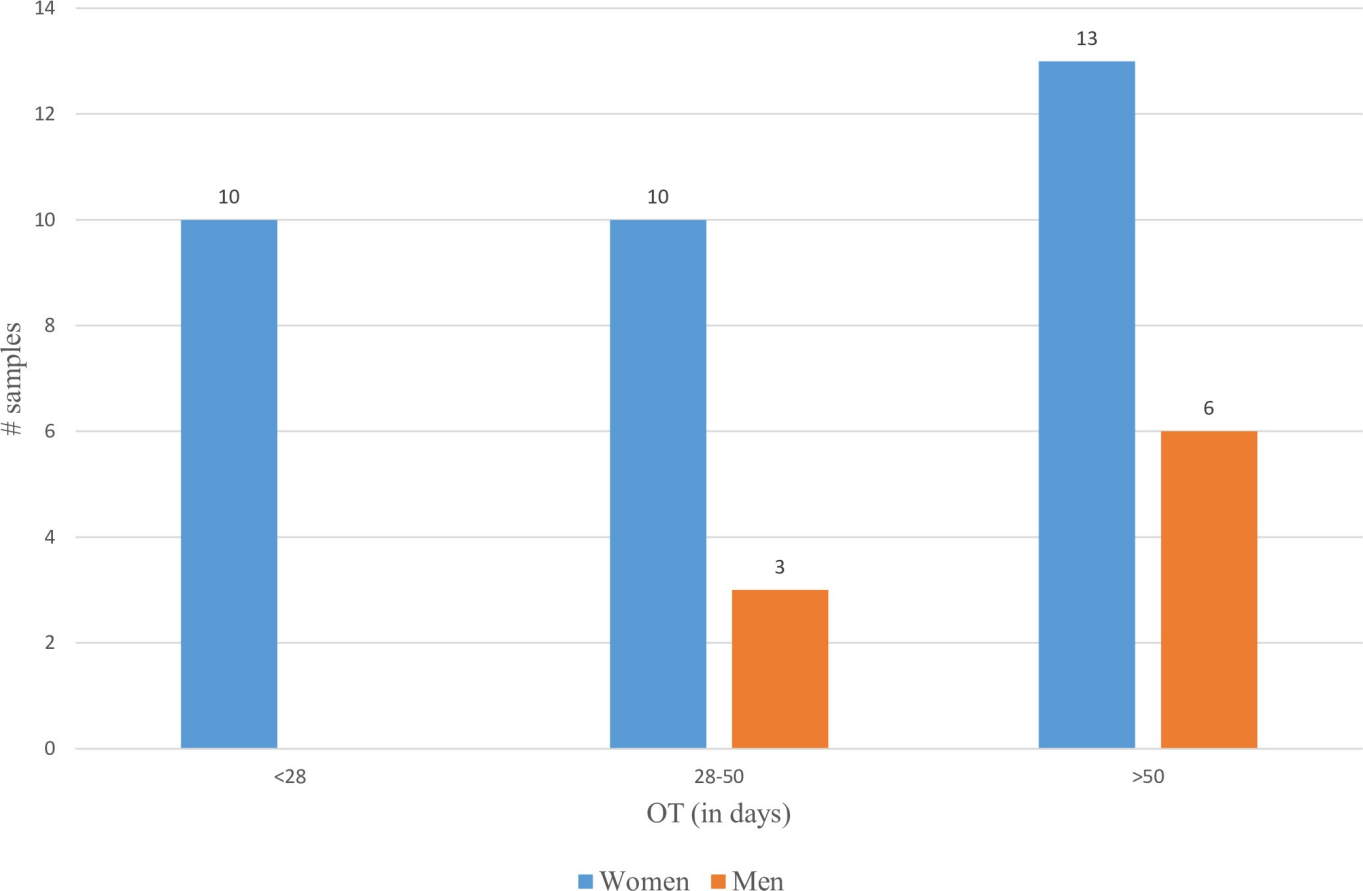
Distribution of participants by Onset of Symptoms to Treatment (OT) time.

Of the 33 female patients sampled, 23 were from Bihar, with equal numbers having early, moderate and late access; while 10 were from Jharkhand, of which half were those with late access. Women from SC communities was predominantly from Bihar and those from ST communities from Jharkhand, in line with the overall distribution of communities in the two states. Seven out of ten women with late access were from ST communities while two of the three deceased were from ST communities and one from OBC community. Of the nine male patients sampled, four were from OBC communities, three from SC and two from ST communities. About a third of the female patients lived 15 km or farther from the nearest primary health centre (PHC), while two-thirds of the female patients were either landless labourers or worked on non-irrigated land of their own. All women with early access, nine out of ten women with moderate access and nine out of thirteen women with late access lived in houses partly or fully made of mud. A conspicuous observation was the abject poverty of most of the participants and their living conditions, such as inadequate warm clothing to protect against the cold weather. Fig 1 gives the distribution of patients by OT.

### Early treatment choices

#### Self-medication

All patients, including those who were deceased, initially treated the symptoms themselves using medicines purchased over-the-counter for varying lengths of time. Men who migrated to cities for work during the early stages of the illness, or had already migrated when symptoms began, resorted to self-medication in the locations in which they had migrated to. In general, men and women with late access self-medicated for up to two months, while men and women with early and moderate access did so for a few days to a few weeks.

“I did not tell anyone for a month, took tablets only”.–WM4

“I bought medicines from the corner shop and took for a month”.–WM5

“I took tablets from the local shop”.–WL13

“In the meantime, I took tablets for fever, from local shops. I thought, ‘It’s only fever; it will become alright’”.–WM2

#### Treatment from the local provider

Unqualified, itinerant providers of care were the first port of call beyond self-medication for almost all participants, regardless of OT. About half of the women with late access and a few women with moderate access sought care from several local providers. The “local doctor” (as they are referred to by the community)–tended to have worked within the community for long enough to have earned their trust as one who provided fairly effective initial care. Patients reported that the local doctor gave medicines on credit, which appeared to be an attractive feature for such a poor population.

“We trust this local doctor and usually go to him. He also treats on credit. And we had no money at the time”.–WM8

“We took him to the local doctor. He is the doctor we trust, and everyone here goes to him. And he treats well. He gave medicines for 10 days”.–SA1

“I took tablets from the local doctor. He would come here every day. We go to this doctor for every illness”.–SA3

“The neighbours said ‘He (the local provider) is a good doctor. Even children get better with his treatment. And we can even pay him later, as he’s a village doctor’”. WE1

“The doctor in the town gave medicines for 10–12 days. For all common illnesses, we go to him, so I went, too”.–WE10

“I went to a local private doctor, after 2 months of fever, along with my husband. For all the illnesses in our family, we go to this doctor. So I went to this doctor thinking my fever will get better (with the treatment he gives).”–WL3

“I decided to show to the local doctor. He was the nearest one and also we could pay him later, so we decided to go to him”.–WL5

“Then we decided to go to a local private doctor. He is popular–many in our village go to him”.–WM6

At the same time, the patients were under no illusion regarding the scope of the local provider’s expertise, and they knew that illnesses often went beyond their ability to treat. Community elders echoed all of these perceptions.

“We thought to first show to a local doctor, and if not better, to go to a better hospital”.–ML1

The local providers who participated in the study were very aware of this trust placed in them, reciprocating it with their being available and accessible, and with what they perceived to be timely referral to higher levels of care. They report that they have suspected VL in patients and have encouraged them to go the government facility for testing and treatment. A few KTS’s attributed the delay in patients reaching government facilities to local providers’ attempt to retain patients. The patients, however, indicated that they switched providers when the treatment did not work.

### Drivers of early treatment choices

#### Vague symptoms

A key driver was the perception that the illness was “mild” or was “just a fever” amongst most of the women with moderate and late access and one woman with early access, as well as in the case of two of the three deceased. They hoped the illness would improve with self-medication or with treatment from the local provider.

“We thought he’ll get better as the fever was mild”.—SA1

“I thought, ‘This is just a weakness, just like that, and I am not ill’”.–WL11

“I thought, ‘This is an ordinary illness; it will get better’”.–WL12

“I waited for 10 days thinking fever will get better with just tablets”.–WL5

“I thought I would get better just like that; it’s only a ‘mild fever’”.–WM4

The majority reported that they were able to continue with their basic physical activities, especially in the initial weeks of the illness. A related factor was their perceived inability to put a finger on what was going on with their bodies; the symptoms did not seem to fit any pattern of illness that they knew of. This fuelled the hope that this could not be a serious illness that warranted financial expenditure.

“I sat at home for another month hoping the fever would get better. I continued to work a little at a time, but I was growing weaker gradually”.–ML5

Seven of the ten women with late access, seven of the ten women with moderate access and relatives of two of the three deceased mentioned this perception. These perceptions are in keeping with the chronic nature of the illness and the insidious onset of symptoms, and it seemed to have helped the patients justify choosing cheaper treatment choices, and the decision to continue working.

“For 15 days, I took local treatment, thinking that this is a mild fever, and will become better”.–WE8.

“In the meantime, I took tablets for fever, from local shops. I thought It’s only fever; will become alright”.–WM2

“After a month (of not taking any treatment), I started taking tablets, thinking this is an ‘ordinary illness’”.–WM9

With tragic hindsight, the wife of a deceased patient remarked that she would advise others “never to consider any illness as mild”.—SA2.

Female patients mentioned the perception that “weakness” is an enduring female health condition and does not require serious attention. Thus, the non-specific symptoms of VL, therefore, were taken to be part of a general fatigue

#### Lack of money

Most women with moderate and late access and two men with late access mentioned that there was no money in the home to afford anything beyond their early treatment choices. The reference to the lack of money implied the perception that their treatment choices were less than ideal, but on the other hand suggested the perceived mildness of the illness did not justify any further expense, especially in such a disadvantaged population. The lack of money, however, appeared to be a deterrent to seeking care at private facilities which were generally perceived as providing a better quality of care (see below), and not necessarily a deterrent to visiting a government facility.

One woman with late access stated that she first thought, “It’s only a fever; it’ll go away on its own. And there’s no money anyway.”—WL3

“I thought this is a ‘mild fever’ and will get well with tablets. Also, I did not have enough money to go to other places.”–WM6

“For about a month, we did not know what to do; we did not have enough money.”–WL8

“I didn’t tell anyone for one month. Money was short. I just took some tablets hoping it would get better.”–ML3

Community members as well as local providers in all locations expressed the view that women do not want to spend their meagre resources for their illnesses, and hence delay seeking care for longer than men.

“When the man is not in the house, the woman does not have access to cash and so she delays.”—PP7

“Women don’t want to leave their household chores and also don’t want to spend money, especially when money is already short”.–FGD10

#### No male guardian at home

Many female patients self-medicated and/or consulted with the local provider as they did not have a male member of the family at home to accompany them outside the home, and especially outside the village. This was mostly because the men had migrated to cities for work, or were outside the home from dawn to dusk. Participants in nearly every interview and discussion, including community elders, local providers and KTS in the study echoed this view. A community elder stated: “The man is not in the house; how can we expect the woman to go to the hospital alone?”(FGD6). A related factor was that the women tended to have no access to cash when the man was not home.

“Women are not able to come when the man is not at home”.–HW8

“They (the women) need permission from the man, and the man may be outside (the home); or there may be no money, and it takes time to arrange for money”.–PP4

“Sometimes the delay could be due to lack of money, or because the man is not at home”.–PP5

“Women also delay because they have to wait for the man to accompany”.–HW6

“Women delay more, as they have to wait for a male guardian to accompany them to the hospital. The men are either not willing or stay in cities for work”.–HW7

“I did not go earlier to this doctor, as there was no male relative to take me for treatment”.–WL1

“For the first two months, my family members decided to get me treated by the local doctor. My husband was in a city for work”.–WL4

#### Low prioritisation of womens health

Local providers reported a pattern of longer delays in care seeking amongst women compared to men, even when the women were aware of the need to seek care. All community groups as well as local providers and government staff opined that women prioritized their household chores over seeking treatment on time.

“Women go for treatment only after finishing all their household duties. That often leads to a delay.”–FGD1

“Women delay because they don’t want to leave their work and come to the clinic.”–PP11

“Women delay more than men, because women have more responsibilities to fulfil.”–HW2

Female patients, as well as community groups mentioned that there is “a lot of work at home”, demanding constant attention from the woman, leaving her with no time to attend to “mild” symptoms. Community groups and local providers also reported that families considered that the woman’s health was not “of such importance” that she should miss household chores to attend to it. One local provider (PP9) explained, “Men have to go outside and earn money. It is important work. Women’s work is not that important.” Thus, women’s work in the home is considered inconsequential, and yet not something to be ignored. One woman with late access reported that she requested her husband to take her for treatment when self-medication did not work, but that her husband ignored her request for some days.

“When it did not get better after some days, I told my husband to take me for treatment. He didn’t pay attention to it”.–WL3

All of these point to a pattern of low prioritisation of women’s health, both by the women themselves and also by their families. These perceptions are echoed in a 2003 study from Bangladesh on care-seeking patterns among women with VL, which found that women try to ignore their illness as long as they can so as to avoid additional expense [17].

#### Combination of drivers

The above factors seem to have worked in combination, and made for potent arguments driving the early treatment choices that the patients made. Self-medication and treatment from the local provider seemed to help patients buy time, in order to arrange for money (often borrowed) or to get a male relative to accompany them in order to move to the next set of treatment choices.

### Subsequent treatment choices

#### Further treatment at private facilities

As the illness progressed, the majority of patients reported having sought care from private facilities and clinics. Most of these facilities are run by qualified providers, and are located in nearby towns, while some participants travelled up to 40 km to seek care at facilities that were popular amongst the local community. In an extreme instance, one woman went to 15 local providers and private facilities before being told to go to a government facility. In all of these instances, these facilities provided treatment for up to a few weeks, after which they ran a host of tests for the participants, including for VL. In most instances, the diagnosis was made at this point, and these facilities encouraged the participants to go to a government facility for further treatment, but in two cases, they attempted to treat it themselves, without success, wasting precious time. In one instance, the private facility told the patient’s family to “sell your land, not just goats, and bring the money for treatment” (ML2), as the illness was a serious one.

#### Tipping points

For most of the patients, diagnosis of VL at a private facility and encouragement of private providers to seek care at a government facility was the tipping point in seeking care at the latter. For a few of them, it was an encounter with a “government health worker” (presumably the KTS) from the local government facility, who had come to their village looking for cases of prolonged fever. A few others reported being noticed by the ASHA, or approaching her themselves, who then accompanied them to the government facility. For a small number, a neighbour or a relative suspected that the illness could be VL, or another serious illness that required that they attend a government facility. There was only one patient in the sample, a woman with early access who did not “shop” for care, but went straight from being identified as a suspected case in her village during a health campaign to seeking care at a government facility.

### Seeking care in government facilities

#### The government facility as an early treatment choice

For most patients, the government facility was not the first, or even the third or fourth option for treatment. This is based on their past experience and perception that these facilities do not have all the tests and treatments available. The latter perception was voiced in all discussions with community elders and in interviews with a few patients.

“Govt hospitals take a long time to treat us. We have to go there again and again and that means more expenditure.”—FGD5

“People generally prefer private hospitals, even though it costs more, because in a govt hospital, you have to stand in line for hours and then the man there gives 1–2 tablets and says ‘That’s it. Done’.” -FGD6

“Although treatment is free in government hospital, it is far off, and often medicines are not available there.”–FGD9

“It’s all good (at the government hospital). But we don’t get all the medicines that are prescribed.”–FGD2

“They (the staff at government hospitals) speak dismissively to us.”–FGD8

A related perception for some was that the government facility is where the poor go for treatment while those who can afford it, go to private facilities, implying that the former is a less-than-ideal choice.

“People go first to govt hospital because we are all poor. If we don’t get better there, we think of going to a private hospital.”*–*FGD7

#### Too early to detect VL?

In a stark departure from the above sentiment, three patients–one man with late access and two women with moderate access–sought care at a government facility within two weeks of the onset of symptoms. However, they were not tested for VL, presumably because of the early stage of the disease at the time means that testing with available tools is not recommended. The male ended up in a series of other facilities before finally returning to the government facility, amassing an OT of 120 days. The women, however, returned to government facilities within about 35 days, but not before going through a round of visits to private facilities. Two other women, one with late access and the other with moderate access reported testing negative for VL at a government facility but went through a series of private facilities before returning to the government facility and subsequently testing positive. None of the above participants recall being given advice on follow-up during their first visit to the government facility.

#### Relief with treatment

Patients vividly recall the dramatic and complete relief from symptoms that they had following treatment. They also recall having had an overall pleasant experience at government facilities where they received treatment. One woman recalled that the facility staff gave her and her family money to get a ride back home.

### Low levels of suspicion of VL

A striking finding in the study was that many patients and their family members did not consider VL as a possibility, even after weeks and months of having the symptoms, and after having rounds of unsuccessful treatment. This appeared to be despite having experienced a case of VL in the immediate or extended family, within the neighbourhood or even having been treated for VL themselves in the past. Others reported that they learnt about VL through awareness activities that were held in their neighbourhood, and could recite the salient features of the disease, and yet did not suspect VL in themselves. In an extreme instance, the husband and mother-in-law of a woman with late access had suffered from VL in the previous 2 years and yet she did not suspect VL in herself, but went from self-medicating to several private facilities and a traditional healer before ending up at the local government facility.

Others who had prior knowledge of VL, including those who have had VL in the family or in their neighbourhood, stated that they would have gone to the government facility earlier and “not run here and there for treatment”, had they gotten a clear indication earlier that they were suffering from VL.

### Awareness regarding VL

Levels of awareness in communities ranged from comprehensive knowledge of causation, transmission, treatment and prognosis, to just knowing that VL is a killer disease. Misconceptions were few and not widely reported, and they include the belief that VL is caused by evil spirits and that it spreads through coughing. Several patient-participants stated that they had not heard about VL prior to falling ill, but picked up the basics from staff at the government facility during treatment. Several non-patient participants stressed the need for creating more awareness, while others felt that public awareness was already high. All patient-participants were confident to encourage others with similar symptoms to seek care at the government facility if symptoms persisted.

## Discussion

### Patterns in care-seeking and drivers of choice

**Fig 2:**
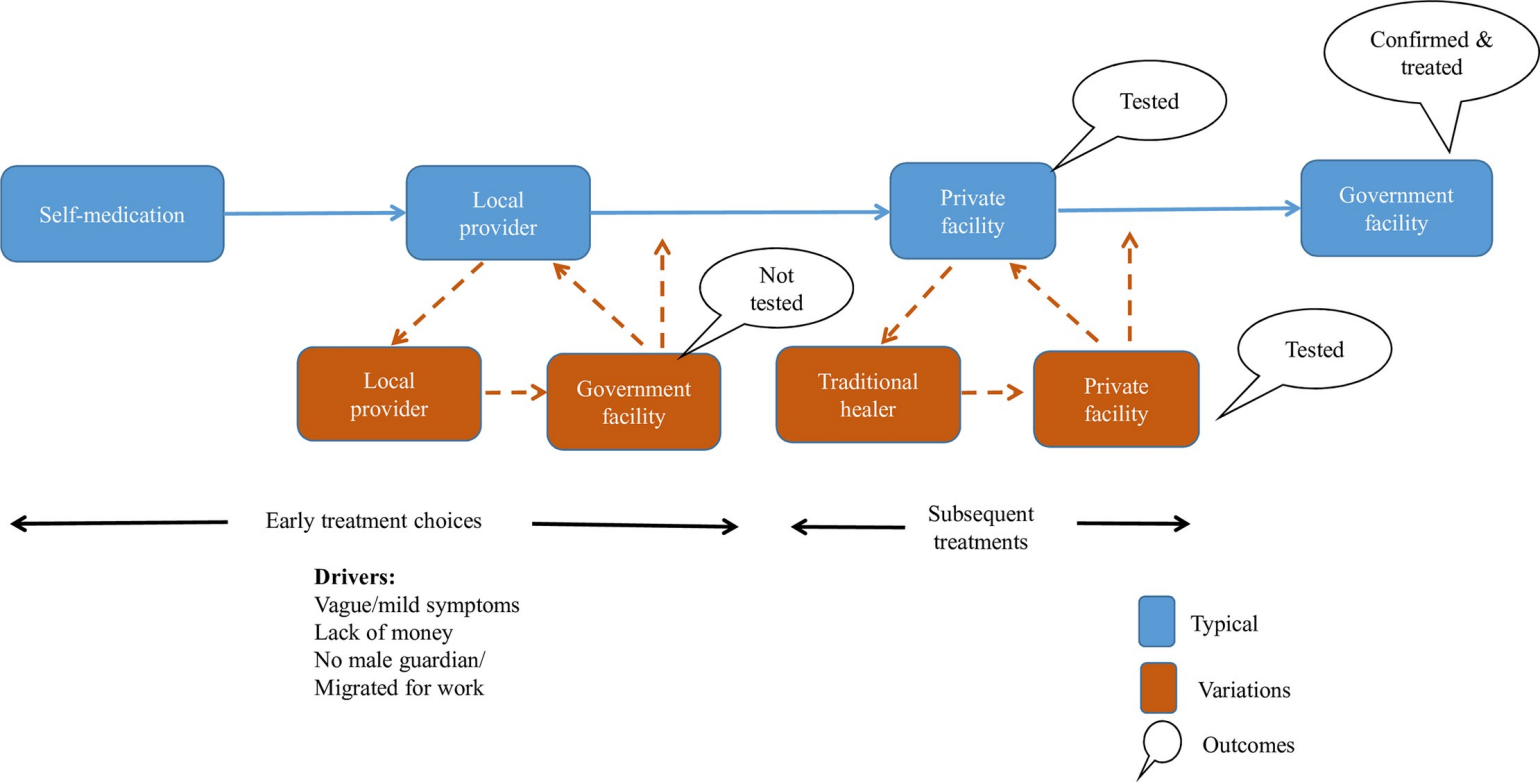
Care seeking patterns: Overall.

The overall care-seeking pattern shows similarities across male and female patients and those with early, moderate and late access: Initial self-medication, followed by repeated visits to or by the local provider, and visits to one or more private facilities, the last of which diagnosed VL and encouraged them to reach a government facility. Perceptions about the severity of the illness, lack of money, not having a male relative in the home and having the male of the household migrating for work drove early treatment choices. These factors also decided how long each of episode of care-seeking would last. This pattern appears to have been more drawn out for women and men with late access, but not for those with early and moderate access, depending on which of those factors were present. Some went to a government facility early on, but were not tested for VL. Fig 2 below depicts this typical pattern, along with some variations.

The government facility is not typically an early choice, but those who did attend one within two weeks of the onset of symptoms were not tested or had a negative test. Without a clear follow-up plan, they went back to treatment shopping. It is unfortunate that patients that reach the public health system in highly endemic districts for VL are not offered advice about the possibility of persistent fever being VL and the important to return for follow up, and it represents significant missed opportunity.

#### Variations within the overall pattern

**Fig 3:**
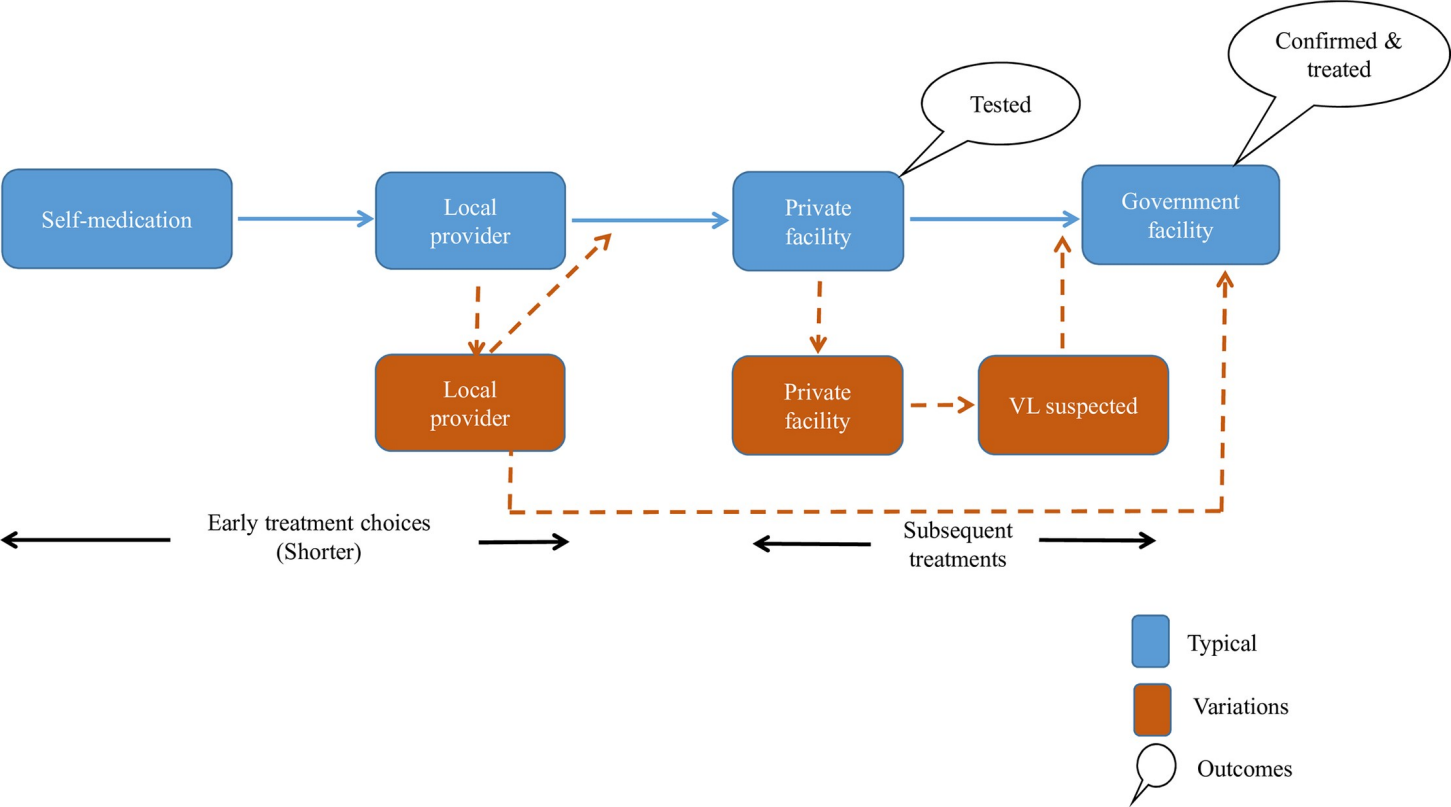
Care-seeking patterns of women with early and moderate access.

Women with early and moderate access and men with moderate access seem to have stayed for a shorter period of time with their early treatment choices and moved on to private providers. Moving to private providers seems to have expedited the chance of diagnosis and encouragement to ultimately attend a government facility in many cases. In some instances, the patients was familiar with another person who suspected VL and guided them to the government facility, thus cutting short what could have been a longer search for cure. Fig 3 below depict the variations for women with early and moderate access.

The low level of suspicion of VL among patients and their families has been a surprising finding considering the breadth of the elimination effort in the highly endemic districts. This is unlikely to be due to patients ignoring the possibility of VL, as these very patients state that they would not have shopped for treatment had they been diagnosed with the disease earlier. Instead, there appears to be an implicit expectation that a potentially fatal disease would have unequivocal and severe symptoms. It also appears that the illness does not stay long in corporate memory, ostensibly due to the insidious onset and non-specific symptoms. These suggests a need to include stronger messages that symptoms of VL can be mild and non-specific.

Unsurprisingly, local providers (‘local doctors’) appear to be a vital part of the local healthcare ecosystem. A study of VL patients in Bangladesh found that 49.5% of patients first consulted a local, unqualified provider [18]. Although these providers are not recognised as health providers, further improving and sensitising them to VL may improve the earlier detection and treatment of cases, while encouraging them to promote the importance of health in women suffering from persistent fever.

The low status of women seems to underlie the other, more immediate drivers of choice. Female patients appear to have inherently accepted the low prioritisation of their health, as is evident in their playing down of their illness more often than men and for longer periods of time, especially when symptoms are non-specific and hence considered “mild”. The widely reported prioritisation of household chores by women seems to have been born out of this low status afforded to them, both by their families as well as society in general. When they do feel the need to seek higher levels of care, the lack of a male family member to accompany them to the government facility contributes to the delay. The concept of “guardian” is integral to the gendered social order of rural India, and is not only socially acceptable but is also considered as a safeguard for the women who do not have the knowledge and tools needed to navigate the marketplace. Therefore, it will be important that messages addressing delayed for VL care-seeking by women to take into account this reality, and emphasise to male decision makers, and community health decision gatekeepers the importance of encouraging early access to care for women presenting with persistent fever and other non-specific signs of disease.

In summary, the early treatment choices of self-medication and local providers are likely to persist. The mild and non-specific symptoms of VL, lack of money, absence of a male guardian, the ease of obtaining ‘trusted’ treatment at the doorstep and a history of unhelpful experiences at the government facility, underlined by a sense of low status for women and their well-being appear to be the layers of perceptions and harsh realities from which women with delayed access have made higher-order abstractions.

### Recommendations to improve care-seeking by women

**Fig 4:**
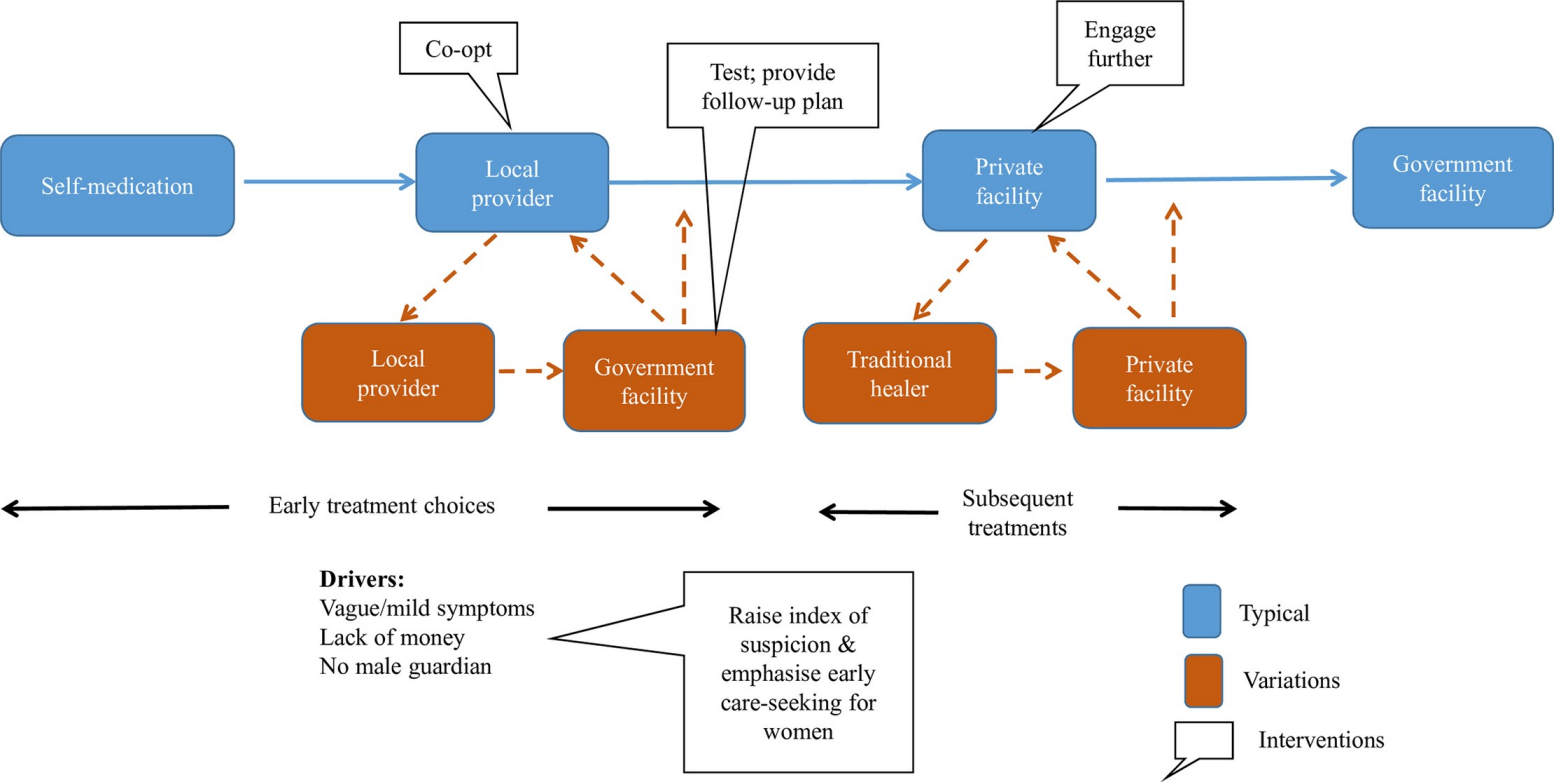
Potential interventions.

At least three options emerge as changeable drivers to expedite care seeking behaviour of women in the short to medium term, as indicated in Fig 4 below.

#### Help raise awareness about suspecting VL strongly when there are vague symptoms, and emphasise the need for early care-seeking for women, through clearer messaging

This study has shown that adequate knowledge about VL and even past experience of the illness in the family and community does not guarantee that VL will be suspected, especially for women. Conversely, whenever VL was suspected, it meant the end of treatment shopping. This shows the need to create and maintain a high level of suspicion for the illness–especially in women who tend to dismiss symptoms as “mild” and not reaching the relatively higher threshold that the community–and they themselves–set for seeking care outside the house. This focus on female perceptions and low prioritisation needs to be relayed to communities, among frontline workers, local providers and private facilities in these highly endemic districts. A qualitative study on the barriers to treatment for VL patients in Sudan found that VL patients perceived the illness to be “dishonest” and “in hiding”, requiring many tests to diagnose it, and highlighted the need to address the barrier caused by these perceptions [19]. A behaviour change intervention study conducted in Bihar and Jharkhand in 2017 found that 77% of households in intervention villages reported that they would encourage anyone with VL symptoms to seek care, compared to 39% in control villages [20]. The focus and messaging in communication and training efforts needs to be adapted to reflect the mildness and non-specificity of symptoms in the early stages of the illness, the importance of maintaining women’s health for the well-being of the entire household and the need for women with symptoms to seek testing at the government facility even when symptoms appear to be mild. Messaging has to factor in the imperative of finding a male relative or trusted neighbour to accompany women to the government facility, if the men in her family are not in the home, and also highlight the fact that women prioritising their wellbeing over household chores is a smart choice in the long run. The impact of delayed treatment in the form of prolonged morbidity and lengthy time being spent unable to conduct activities of daily living should be emphasised.

#### Reduce missed opportunities in government facilities

This study has shown that patients continue to slip through the very system that aims to reduce the time to treatment, albeit largely due to the lack of adequate tools to make a diagnosis of VL in under 2 weeks of onset of symptoms (this is the earliest point that a patient should be tested with the rapid diagnostic test for VL). It also takes a lot more effort and time to bring such a patient back into the system, once the patient has been turned away without the diagnosis made, and as such it is important that practitioners are trained on how to address the ‘risk’ of VL in patients presenting earlier than 2 weeks, through appropriate counselling and follow up in case symptoms persist. Such near-misses also reinforce the perceptions about the quality of care in government facilities. Clinicians need to maintain strong suspicion for VL especially in the face of non-specific symptoms, and protocols revised to include a follow-up plan for those who test negative or who do not qualify for a test.

#### Sensitise informal and formal private practitioners on both VL and gender barriers

The study has shown a clear trend of informal and formal private practitioners encouraging patients they diagnose with VL to attend government facilities. It has also shown that local informal practitioners are much closer and more trusted by women than the government health facilities; as such they can be co-opted to play a major role in emphasising and encouraging women with symptoms of VL to access early and appropriate care. These constitute a low-hanging fruit in the effort to reduce delay for female patients. The programme should aim for wider coverage in sensitizing private providers, and increase the frequency of contact, with the objective of ensuring that patients are referred as soon as VL is suspected or diagnosed.

### Limitations

The study has some important limitations that need to be considered. Primarily, the sampling frame was taken from the national VL surveillance register, which means that patients that were either not reported or not recorded in the register, such as those receiving diagnosis and treatment entirely in the private sector, were not considered in the sampling frame–although recent studies show that underreporting is low [2]. Secondly, the study was limited to high endemic blocks, where presumably the knowledge and awareness of VL is higher than in moderately and lower endemic area, where the results of this study cannot be extrapolated. Finally the small sample of male patients makes comparative analysis and thematic saturation for male patients difficult.

## Supporting information

S1 Checklist STROBE checklist. (DOC)

S1 Table Fulfilment of selection criteria for selected blocks. (DOCX)
