# Supplementary material for: “It’s just a fever”: Gender based barriers to care-seeking for visceral leishmaniasis in highly endemic districts of India: A qualitative study
Source: PLoS Negl Trop Dis. 2019 Jun 27;13(6):e0007457. doi: 10.1371/journal.pntd.0007457 (PMC6597040; doi:10.1371/journal.pntd.0007457)
Supplement: S1 Table — (DOCX) [file pntd.0007457.s002.docx]

**Supplementary table 1:**

| **State** | **District** | **Block** | **# cases per 10,000 population in 2016** | **Change in incidence between 2015 and 2016** | **Median OT among registered cases in 2017** | **# cases with OT >50 in 2017** |
| --- | --- | --- | --- | --- | --- | --- |
| Bihar | E Champaran | Madhuban | 2.21 | Increasing | 30 | 8 |
| Bihar | E Champaran | Chakiya | 1.18 | Increasing | 45 | 2 |
| Bihar | Sitamarhi | Dumra | 1.47 | Increasing | 30 | 2 |
| Bihar | Sitamarhi | Pupri | 1.66 | Increasing | 38 | 2 |
| Bihar | Gopalganj | Gopalganj Sadar | 1.85 | Increasing | 50 | 10 |
| Bihar | Gopalganj | Bharauli | 2.67 | Increasing | 43 | 1 |
| Bihar | Araria | Raniganj | 1.92 | Decreasing | 45 | 9 |
| Bihar | Araria | Forbesganj* | 1.24 | Decreasing | 40 | 0 |
| Jharkhand | Dumka | Gopikander | 5.6 | Increasing | 60 | 5 |
| Jharkhand | Pakur | Maheshpur | 5.1 | Increasing | 30 | 10 |
| Jharkhand | Godda | Boarijore | 5.0 | Decreasing | 30 | 14 |
| Jharkhand | Sahibganj | Mandro | 3.2 | Decreasing | 30 | 1 |

*selected for logistical reasons
